# Promoting faster pathways to surgery: a clinical audit of patients with refractory epilepsy

**DOI:** 10.1186/s12883-019-1255-0

**Published:** 2019-02-19

**Authors:** Virginia Mumford, Frances Rapport, Patti Shih, Rebecca Mitchell, Andrew Bleasel, Armin Nikpour, Geoffrey Herkes, Amy MacRae, Melissa Bartley, Sanjyot Vagholkar, Jeffrey Braithwaite

**Affiliations:** 1 Australian Institute of Health Innovation, Faculty of Medicine and Health Sciences, Macquarie University, Sydney, Australia; 2 University of Sydney, Sydney, Australia; 3 Westmead Hospital, Westmead, Australia; 4 Royal Prince Alfred Hospital, Sydney, Australia; 5 Royal North Shore Hospital, Sydney, Australia; 6 Primary Care & Wellbeing, Faculty of Medicine and Health Sciences, Macquarie University, Sydney, Australia

**Keywords:** Refractory epilepsy, Neurosurgery, Patient pathways, Surgical assessment, Clinical audit

## Abstract

**Background:**

Individuals with epilepsy who cannot be adequately controlled with anti-epileptic drugs, refractory epilepsy, may be suitable for surgical treatment following detailed assessment. This is a complex process and there are concerns over delays in referring refractory epilepsy patients for surgery and subsequent treatment. The aim of this study was to explore the different patient pathways, referral and surgical timeframes, and surgical and medical treatment options for refractory epilepsy patients referred to two Tertiary Epilepsy Clinics in New South Wales, Australia.

**Methods:**

Clinical records were reviewed for 50 patients attending the two clinics, in two large teaching hospitals (25 in Clinic 1; 25 in Clinic 2. A purpose-designed audit tool collected detailed aspects of outpatient consultations and treatment. Patients with refractory epilepsy with their first appointment in 2014 were reviewed for up to six visits until the end of 2016. Data collection included: patient demographics, type of epilepsy, drug management, and assessment for surgery. Outcomes included: decisions regarding surgical and/or medical management, and seizure status following surgery. Patient-reported outcome measures to assess anxiety and depression were collected in Clinic 1 only.

**Results:**

Patient mean age was 38.3 years (SD 13.4), the mean years since diagnosis was 17.3 years (SD 9.8), and 88.0% of patients had a main diagnosis of focal epilepsy. Patients were taking an average of 2.3 (SD 0.9) anti-epileptic drugs at the first clinic visit. A total of 17 (34.0%) patients were referred to the surgical team and 11 (22.0%) underwent a neuro-surgical procedure. The average waiting time between visit 1 to surgical referral was 38.8 weeks (SD 25.1), and between visit 1 and the first post-operative visit was 55.8 weeks (SD 25.0).

**Conclusion:**

The findings confirm international data showing significant waiting times between diagnosis of epilepsy and referral to specialist clinics for surgical assessment and highlight different approaches in each clinic in terms of visit numbers and recorded activities. A standardised pathway and data collection, including patient-reported outcome measures, would provide better evidence for whether promoting earlier referral and assessment for surgery improves the lives of this disease group.

**Electronic supplementary material:**

The online version of this article (10.1186/s12883-019-1255-0) contains supplementary material, which is available to authorized users.

## Background

Epilepsy is a common neurological condition with a lifetime prevalence in Australia of 3–3.5%, equating to over 250,000 Australians, or 1 in 25 adults [1]. The World Health Organization estimates an incidence rate of 2.4 million people per year, and prevalence rates for people needing treatment for epilepsy of 0.4–1% [2]. Seizures can often be controlled using anti-epileptic drugs (AEDs), but for approximately 30% of people with epilepsy, seizures are not controlled despite the use of two or more AEDs, and refractory epilepsy, a severe and debilitating condition, is then diagnosed [3]. Individuals who have refractory epilepsy may be suitable for surgical treatment, especially where a single region of the brain cortex is responsible for generating seizures (i.e. focal epilepsy) [4, 5]. The main evidence supporting surgery has come from a randomized controlled trial of 80 patients with temporal lobe epilepsy which indicated that surgery reduced the incidence of seizures versus medical treatment [6]. These findings prompted the development of a practice parameter in the United States to recommend earlier referral for surgical review for patients with temporal lobe seizures [7, 8]. A later study showed that surgery improved quality of life outcomes. However, the sample size was small and none of the patients became seizure free [9]. A 2015 Cochrane review suggested that the level of evidence for surgery versus medical treatment was inconclusive [5]. Despite these finding, the impact of continuing seizures on patients and the healthcare system, while other types of treatment options are trialed [10, 11], has resulted in further calls for speedy surgical intervention [12]. Surgery in refractory epilepsy is not without risk, however, and while the risk of serious complications is estimated to be below 1% for temporal lobe surgery [13], a paediatric study has reported a serious adverse event rate of 33%, despite a significant increase in freedom from seizures and better quality of life in the surgical versus medication treatment arms [14].

Nevertheless, these risks need to be weighed against the considerable burden of this disease [15, 16]. Patients with refractory epilepsy have to deal with the impact of not being able to drive and the effect of the disease on work and social activities, while individuals with epilepsy, including refractory epilepsy, have a higher rate of mental health co-morbidities than the general population, including depression and suicide [15, 17, 18]. Patients with continuing seizures also face possible brain damage [10], a higher incidence of sudden death [19], and side effects from lengthy anti-epileptic drug regimens [8].

Preparation for surgery is not simple, and patients with refractory epilepsy undergo a detailed pre-operative work-up to determine the type and location of seizure foci in order to assess their suitability for surgery [20]. A key element of this is the use of a video recording of the seizures with a simultaneous electro-encephalogram (EEG). More invasive intra-cranial EEG recordings may also be required to isolate the site of the seizures and may be combined with different neuro-imaging techniques to determine the exact area for surgical resection [20]. Psycho-social testing is also recommended, in order to determine suitability for surgery and to aid decision making for both clinicians and patients [4].

The pathway to surgery involves referral to a surgical team, surgery, and post-operative visits, resulting in an extensive process of multiple medical assessments that can be stressful and can create both physical and mental strain for patients [21]. Consultation, testing and ongoing assessment have also led to concerns that patients are facing considerable delays before arriving at surgical intervention, and that some of these delays are resulting in surgery being abandoned [22, 23]. To address these concerns and consider the issue of delay to surgery in more detail, rather than concentrating on surgical outcome per se, the study focussed on assessing patients’ clinical pathways through care from drug treatments to pre-operative tests, including the timing of specialist visits. By so doing the study team aimed to examine patient journeys through two Tertiary Epilepsy Clinics (TECs) in New South Wales (NSW), Australia and build a more comprehensive picture of contemporary approaches to streamlining services [24].

## Methods

A retrospective audit was undertaken to collect longitudinal data from a clinical record review of 50 patients attending two TECs (25 patients in each clinic) based within two publicly-funded teaching hospitals. These clinics comprise two of the three adult TECs within the NSW Epilepsy Referral Network and were selected for their critical role in managing refractory epilepsy patients’ treatment and care, including surgical intervention. Ethics approval for the study and for a waiver of consent for participants was obtained from the North Sydney Local Health District Human Research Ethics Committee (HREC/17/HAWKE/22).

The clinical record review included patients with refractory epilepsy who were considered potentially suitable as surgical candidates, and who had an initial clinical appointment at either of the two TEC sites in 2014. Patients were deemed to have refractory epilepsy if trials of at least two appropriate and tolerated AED schedules, either as monotherapy or in combination, failed to achieve freedom from seizures [3]. The first 25 patients in each clinic who met these inclusion criteria were selected. The dates were chosen to ensure that the patient cohort could provide a comprehensive clinical record from first TEC assessment to either a surgical or non-surgical treatment outcome, while data were collected from patients’ clinical records for up to a maximum of six clinic visits between 2014 and 2016.

The clinical records were reviewed by a clinical research nurse in each clinic between June and October 2017. De-identified data were extracted using a purpose-designed, online audit tool on the *Qualtrics* Survey platform [25]. The platform improved the management of clinical data, and the transfer of sensitive patient data in a secure and de-identified manner. The audit tool was developed by the research team and senior neurology consultants and clinical nurse leads in each clinic. The audit tool included questions relating to: patient demographics, type of epilepsy, drug treatment, changes to drug treatment during clinic visits, and content of discussions surrounding surgery (Additional file 1).

Due to the importance of mental health co-morbidities in refractory epilepsy, a number of patient-reported outcome measures were included in data collection. These measures included the Neurological Disorder Depression Inventory for Epilepsy (NDDIE) [26], and the Generalized Anxiety Disorder screening tool (GAD7) [27]. NNDIE is a six-item, self-reported questionnaire designed to detect depression in patients with epilepsy. The threshold values for indicating a major depressive episode in these patients is country-specific and range from above 13 (Italy) to above 16 (Germany) [26]. The GAD7 is a self-reported tool comprising seven items that has been validated for identifying generalized anxiety. Further mental health evaluation is recommended for patients with a GAD7 score above 10 [27, 28].

Once full datasets had been collected for all 50 patients, de-identified results from the audit tool were made available to the research team, downloaded from the *Qualtrics* secure database and analyzed using *Stata* (version 15.1) [29]. After compiling the descriptive statistics, two-tailed T-tests were used to determine whether there was a statistical difference in means between patient and clinic-level outcomes (using a 5% significance level), and a correlation analysis was undertaken to investigate the relationship between the main variables.

## Results

### General demographics

The mean age of the patients in this study was 38.3 years (SD 13.4, median 38.5) and patients had been diagnosed with epilepsy for an average of 17.3 years (SD 9.8, median 16.5). All patients were referred to the TECs due to refractory epilepsy. Eighty eight percent of patients (44/50) had a principal diagnosis of focal epilepsy, and generalized epilepsy was diagnosed in 2 patients. Of the four remaining patients, two were considered psychogenic in nature, and two were related to developmental delay and traumatic brain injury. Employment was not noted for 15 patients at the first visit and of the remaining patients, 16 people were in full-time employment, seven were unemployed and not looking for work, and six were students. Two patients over the legal driving age converted from being ineligible to eligible to drive, post-surgery. The remaining nine surgical patients remained ineligible to drive during the study period (Table 1). Analysis of patient postcodes identified that 50% of patients were located outside the greater Sydney area, with two patients travelling ‘interstate’ for appointments.

**Table 1:** Patient characteristics at first clinic visit to two Tertiary Epilepsy Clinics in New South Wales in 2014

Patient Demographic Summary across both clinics		
	Total	
Mean patient age at first visit (years)	38.3	
*SD*	*13.4*	
Mean years since diagnosis	17.3	
*SD*	*9.8*	
Whether eligible to drive	**n**	**%**
Yes	6	12.0
No	34	68.0
Not stated	10	20.0
Employment status (first visit)		
Disabled	2	4.0
Employed full time	16	32.0
Employed part time	0	0.0
Retired	1	2.0
Student	6	12.0
Unemployed looking for work	3	6.0
Unemployed not looking for work	7	14.0
Not stated	15	30.0
Marital status (first visit)		
Divorced	3	6.0
Married/de facto relationship	26	52.0
Separated	1	2.0
Single	17	34.0
Not stated	3	6.0
Diagnosis		
Focal epilepsy	44	88.0
Generalised epilepsy	2	4.0
Other	4	8.0

### Number and timing of visits

The average number of clinic visits per patient was 4.3 out of a possible total 6, giving a total 217 separate patient encounters over the study period. Patients in Clinic 1 had an average of 5.5 (SD 0.9) visits during the study period, which was significantly higher (*p* < 0.001) than the average number of visits in Clinic 2 (3.2 visits, SD 1.8). Patients having surgery also had a higher than average number of visits across both clinics than those not having surgery (5.3 versus 4.1). After undergoing pre-operative testing, patients were usually referred to the surgical team before a final decision was made regarding surgery. However, surgical team visits were not included as part of the data collection, but identified instead at the neurology outpatient visit, where a decision was made for surgical referral. The average time to referral to the neurosurgical team at both clinics was 38.8 weeks from the first visit (SD 25.1), and to the first post-operative visit was 55.8 weeks (SD 25.0). The time from first visit to a decision to refer the patient to surgery was higher for patients who did not undergo surgery during the study period (8/17) than for the 9/17 patients who did undergo surgery following referral: 50.2 weeks (SD 28.7) versus 28.6 weeks (SD16.9), but the difference was not significant. For the patients undergoing surgery, the average time between last pre-operative visit and first post-operative visit was 20.4 weeks (Table 2).

**Table 2:** Patient visit data: timing and number of visits by patient and clinic

Timing and number of visits by patient and clinic				
Number of visits by patient	Clinic 1	Clinic 2	Total	%
One visit	0	5	5	10%
Two visits	1	7	8	16%
Three visits	0	2	2	4%
Four visits	2	5	7	14%
Five visits	4	2	6	12%
Six visits	18	4	22	44%
Mean weeks from 1st visit to surgical referral	35.9	42	38.8	
*SD*	*8.8*	*3.4*	*25.1*	
*N*	*9*	*8*	*17*	
Mean weeks from 1st visit to post-op visit	57.2	54.5	55.8	
*SD*	*31.1*	*21.7*	*25.0*	
*N*	5	6	11	
Mean weeks from pre-op to post-op visits			20.4	
*SD*			*9.2*	
*N*			*11*	

**Fig. 1:**
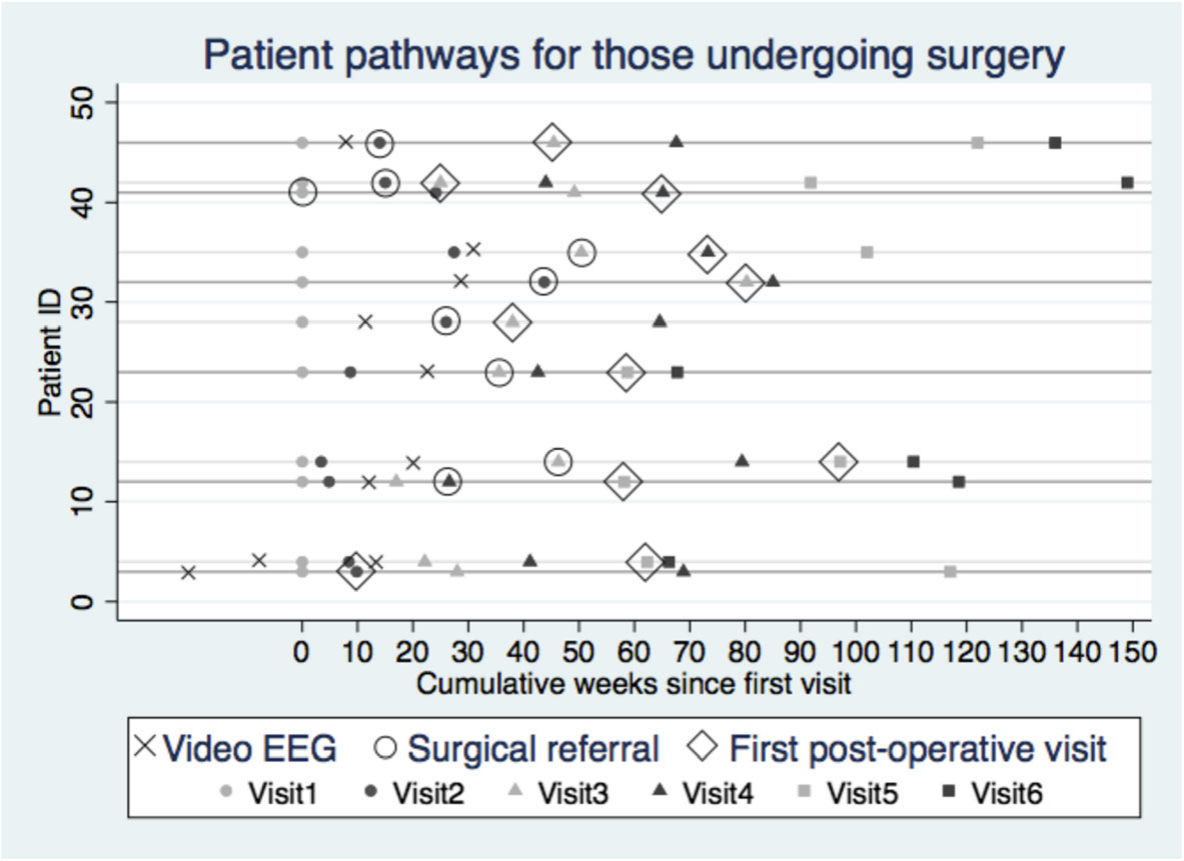
Clinic activity timelines for surgical patients

Timelines for the eleven patients with a post-operative visit during the study period are shown in Fig. 1 below, which illustrates the pattern and timing of visits, including timing of video EEGs, decisions to refer patients for surgery, and post-operative visits. In some patients, video EEGs were performed before presenting to the clinics, and two patients had repeat video EEGs during the study period. Figure 1 indicates that the patient represented by the top line of the graph underwent a video EEG between visits 1 and 2, was referred to the surgical team on visit 2, returned to the clinic for a post-operative assessment on visit 3, and had a further three visits within the study period. A more detailed graph for all patients is shown in the Additional file 2.

### Surgical management of epilepsy

Of the 50 patients in this study, 11 underwent a cycle of pre-operative assessment, referral to the surgical team, surgery, and a post-operative visit during the two-year audit period. These 11 patients had an average age of 36.9 years. Surgical management was discussed at a total of 113 encounters and at 64% (54/85) of all visits for the 17 patients referred for surgery. Records of discussions of the adverse events of surgery were noted only in patients from Clinic 1, and for eight out of nine patients referred to the surgical team, and three out of five patients who underwent surgery. A decision not to proceed to surgery was noted for eight patients after an average of 2.5 visits, and for one patient after referral for surgery.

### Tests and pre-operative work up

Blood tests for monitoring anti-epileptic agents were conducted at 5.5% (12/217) of visits and electro-encephalograms (EEGs) were ordered on 23.0% (50/217) of visits. This is in addition to the pre-operative workups (which included video EEG tests) that were conducted on 24 patients that did not undergo surgery in addition to ten of the 11 patients who did have surgery during the study period [20]. Five patients had completed their video EEGs prior to their first appointment, two of whom had surgery during the study period.

### Drug treatment of epilepsy

Patients were taking an average of 2.3 AEDs (SD 0.9, median 2) on the first visit, with five patients on four drugs, and ten on one drug. At the time of referral to surgery, patients were taking an average of 2.82 AEDs, which is higher than the average of all patients on visit 1, but the difference was not statistically significant. Anti-epileptic therapy was actively managed during the visits, with one drug changed for 16 patients (32%) in visit 1, and a total of 101 drugs changes were made for 37 patients on 42% of visits overall. The most frequently changed drugs were Lacosamide (16.8% of changes) and Sodium Valproate (15.9% of changes). Drug dosage was also actively reviewed with one or more changes to drug doses for 13 patients on the first visit and drug doses changed on 37% of all visits (comprising a total of 87 drug doses). The most commonly used drugs on the first visit were Levetiracetam (*n* = 23), Carbamazepine (*n* = 20) and Lamotrigine (*n* = 16). A number of agents such as Vigabatrin, Gabapentin, Perampanel and Piracetam were not being used at the time of the first visit but were added in later visits. Analysis of the top three drugs most commonly used at the time a decision was made to refer to the surgical team showed the same drugs: Carbamazepine (*n* = 10), Lamotrigine (*n* = 8) and Levetiracetam (*n* = 7) (Table 3).

**Table 3:** Drug treatment of all refractory epilepsy patients at first TEC clinic visit

Summary of anti-epileptic drugs taken by patients on first TEC clinic visit	
Anti-epileptic drug	Number of patients on each drug at the first visit
Levetiracetam	23
Carbamazepine	20
Lamotrigine	16
Valproate	12
Topiramate	11
Lacosamide	7
Zonisamide	5
Clobazam	5
Oxcarbazepine	5
Phenytoin	5
Clonazepam	2
Piracetam	0
Perampanel	0
Vigabatrin	0
Gabapentin	0
Nitrazepam	0

At the first post-operative visit the average number of drugs taken by the 11 patients who had surgery was 2.27 (SD 0.90) versus 2.18 (SD 1.32) for these same patients at their first visit. Table 4 details the drugs taken by these 11 patients and illustrates the active management of these patients. The drugs recorded on the first visit are shown above the drugs recorded in the first post-operative visit for each patient. For example, Patient a was on Levetiracetam on the first visit, with Zonisamide and Oxcarbazepine added by the first post-operative visit. Patient k was on Levetiracetam and Carbamazepine on both visits with no changes to their treatment pre-, and post-surgery.

**Table 4:** TEC prescribing practices for patients pre- and post-surgery

Changes in AEDS prescribed pre- and post-surgical intervention													
		Surgical patients											
		**a**	b	**c**	d	**e**	f	**g**	h	**i**	j	**k**	Total
Lamotrigine	Visit 1				X	**X**		**X**		**X**	X		**5**
	1st post-op visit			**0**	0	**0**		**0**		**0**	0		**6**
Levetiracetam	Visit 1	**X**	X						X		X	**X**	**5**
	1st post-op visit	**0**	0						0		0	**0**	**5**
Carbamazepine	Visit 1		X		X	**X**	X				X	**X**	**6**
	1st post-op visit			**0**	0		0				0	**0**	**5**
Zonisamide	Visit 1						X			**X**			**2**
	1st post-op visit	**0**					0	**0**		**0**			**4**
Lacosamide	Visit 1						X						**1**
	1st post-op visit		0				0						**2**
Clobazam	Visit 1				X					**X**			**2**
	1st post-op visit							**0**					**1**
Oxcarbazepine	Visit 1												**0**
	1st post-op visit	**0**											**1**
Clonazepam	Visit 1						X						**1**
	1st post-op visit						0						1
Total number of drugs (Visit 1)		1	2	0	3	2	4	1	1	3	3	2	
Total number of drugs (1st post-op)		3	2	2	2	1	4	3	1	2	3	2	
Number of drugs changed		2	1	2	1	1	0	2	0	1	0	0	

*X denotes AED regimens for each patient at first clinic visit*

*0 denotes AED regimens for each patient at first post-operative clinic visit*

### Quality of life and patient-reported outcome measures

In total, 22 patients from Clinic 1 were assessed on the first visit using the NNDIE, with 12 of these patients showing a score of 13 or above, and five showing a score above the higher threshold level of 16. The mean scores on visit 1 were 12.6 (SD 4.3). The mean scores for the 21 patients tested on the GAD7 in Clinic 1 in the first visit were 6.5 (SD 4.8), and four patients had scores above a referral level of ten. Only one patient was tested post-operatively.

### Correlation analysis

A multivariate correlation analysis indicated a positive correlation between the scores for the two mental health tools (0.60). There was also a positive relationship between being married and being employed (0.52) and being eligible to drive and age (0.56). However, none of these correlation co-efficients were significant at the 5% level when we applied a Bonferroni correction for the number of variables.

## Discussion

These results present a detailed picture of patient pathways to surgery in NSW, Australia, during the study period. The strength of this study lies in it being the first study of its kind to undertake a detailed analysis of out-patient activity in two NSW TECs, with two-year data capture and analysis outlining the prescribing activity and pre-operative assessment for two refractory epilepsy patient cohorts. It is also unique in its application of a newly created clinical audit tool, linked to the *Qualtrics* Survey Platform, which was designed by the study team to enable extensive insights to be gained into patient pathways, temporal aspects of care provision, AED use, and treatment decisions around the complex process of assessment and build-up to surgery for refractory epilepsy patients, and will form the basis of future research.

The 11 patients who underwent surgery during the study period had an average age of 36.9 years (SD 12.8), and a diagnosis of epilepsy for an average of 18.5 years (SD 10.7). These results are similar to a 2010 US study of 102 patients which showed a mean age of 37.0 (SD 11.8) years, and mean years since diagnosis of 18.6 (SD 12.6) years [30]. The mean waiting time from the first visit to surgical referral for the patients in our study was 38.8 (SD 16.9) weeks, with an additional 17.2 weeks between referral to the surgical team and the first post-operative visit. This compares favorably to a 2017 Canadian and Mexican comparative study which showed the average waiting times from the first epilepsy center visit to surgery  of 111.4 weeks (*n* = 72) in Canada, and 182.8 weeks (*n* = 81) in Mexico, versus 55.8 weeks in our study [31].

Our results are conservative as we were assessing the time to the first post-operative visit rather than the actual surgery date but comprise a smaller sample size of 11 post-operative patients. The results for the age at presentation to the clinic and years living with epilepsy were similar to the Canadian and Mexican study: patient age was 36.7 and 37.4 in Canada and Mexico, respectively, versus 36.9 in our study; and years with epilepsy were 20.2 and 27.4 in Canada and Mexico respectively, versus 18.5 years in our study. The literature indicates that delays in surgical treatment are mainly due to delays in referral to specialist clinics, rather than delays in assessment and the decision to proceed to surgery once under the care of the clinic [30, 31].

The delays from community neurologist consultation to an initial TEC specialist referral can be related to a number of factors including: the degree to which the neurologist is familiar with an individual patient’s diagnosis, knowledge of drug and therapeutic regimes, and the extent of understanding of a patient’s specialist care needs. A US study highlighted the importance of a patient’s health insurance status including differences in the degree to which healthcare insurance covers treatment and drugs needed [32]. However, what is striking, is that despite differences in health care funding, gross domestic product, and geographical dispersion between the four countries (Australia, Canada, the United States, and Mexico), time from initial diagnosis of epilepsy to referral to a specialist clinic is similar [31, 32].

In addition to the delays mentioned above, the centralised nature of TEC specialist clinics makes access more difficult for patients living in rural locations (in the case of this study cohort, 50% of patients lived outside the greater Sydney area) especially given the range of tests needed in the pre-operative workup phase. However, attending clinics that are centralised and therefore part of a major hub for clinical activity has a number of advantages: 1) it enables patients to share experiences with other patients in similar circumstances; 2) it enables patients to access more individualised care [21]; and 3) it ensures patients receive information and care from specialists dealing in refractory epilepsy on a daily basis and  familiar with diagnosis and care pathways. Consequently, while more could be done to streamline services once patients arrive at a TEC from a rural location, and ensure fewer delays once patients are within the TEC care system, there is much to be gained from accessing services centrally and taking advantage of the specialist advice and guidance available.

Discussions with clinical team members in community neurology clinics suggested that mechanisms to improving referral times to the clinics should take account of: TEC specialists attending community clinics to ensure greater community-TEC collaboration and shared care practices; the provision of advice on treatment options to be placed on patient advocacy websites (e.g. Epilepsy Action Australia); and training for community-based neurology clinicians in the use of validated online tools to assess suitability for surgery [33].

The results of this study give a unique insight into the active management of these patents in terms of both drug selection (at least one drug was changed at 42% of all visits), and drug dosage (at least one drug dose was changed at 37% of all visits). While a recent study has shown that adding new AEDs stopped seizures in 13.9% of refractory epilepsy patients, and reduced seizures in 38.2% [34], it is difficult to make a direct comparison with our study, as although 14 patients became seizure-free during the study, eight of these had surgery, which may have impacted their seizures, and five did not have any new drugs added.

The main limitation of our study was the modest number of patients reviewed (*n* = 50) which limits statistical comparison. However, this can be seen in the context that, on an annual basis, the number referred for surgery over the two years of the study (*n* = 17) represents 13% of all patients undergoing refractory epilepsy surgery in NSW, Australia, using 2012–2016 data [11]. The sample size, especially those undergoing surgery (*n* = 11), also makes it difficult to report statistically significant differences in location of seizures or types of surgery undertaken. We therefore did not include these factors in this study which was designed to assess the different patient pathways, referral and surgical timeframes, and surgical and medical treatment options. However, given the importance of these factors in determining the impact of surgery, we will address this in in future studies that follow a larger cohort of patients through the surgical assessment pathway.

A clinical audit tool approach has its limitations in that it can only measure activities as recorded in patients’ clinical records, and not, for example, phone calls that act as additional points of contact with patients. We noted differences in data records across the clinics, including; the discussion of adverse events, the collection of patient-reported mental health measures, and the number of visits. The latter point may be related to the referral catchment area for each clinic, in terms of availability and specialty of local neurology services. The differences in other data capture that impact data assessment may be due to a difference in recording style and specialist interest in non-clinical patient outcome measures. A consistent approach to recording patient-reported outcome measures and clinical data reporting may help inform evidence-based clinical decision-making during assessment for surgery.

A further limitation of the study was the disparity of the number of visits, between one and six, during the study period, which affected our ability to use panel data techniques to comparatively evaluate and analyze results across visits due to the censored nature of the data. However, we have detailed the results at the first visit (where all patients attended) and then at critical time-points throughout the patient journey, such as referral to surgery and the post-operative visits, as these milestones are not related to the visit number. In recording these differences in attendance rates across the two clinics we have shown that a number of useful insights regarding consulting patterns and patient pathways can be obtained, and a strength of this study was its ability to highlight that regardless of variability, similar numbers of patients at each clinic underwent surgical intervention.

The results show that only three patients had more than one post-operative visit within the study period, and that only one patient underwent post-operative mental health assessment. Increasing the length of study follow-up would provide a more detailed understanding of the effects of surgery on patient quality of life, mental health, employment, ongoing relationships and the ability to drive. An expanded program of research, to capture a longer post-surgical follow-up, would also provide further evidence as to whether the number and type of side effects from anti-epileptic drugs is reduced after surgery, and whether patients with significant indicators of mental health co-morbidities are referred for further mental health assessment and treatment [17].

## Conclusion

The results of this study have helped to contextualize the NSW, state-wide, epidemiological data regarding acute care encounters [11], in addition to adding vital detail to the in-depth qualitative interviews and observations that this team has already undertaken with refractory epilepsy patients undergoing surgery during 2016 [21]. Clinical record audit is an important tool for analysing patterns of patient care received at health system encounters, especially for chronic conditions, and when the burden of disease for patients is so extensive as is the case with refractory epilepsy patients [15]. Building a better evidence-base at the critical stage of a patient’s entry into a TEC, and for their subsequent pathway towards surgical assessment and intervention, can provide valuable insights into whether the promotion of earlier referral and assessment for surgery improves the lives of this disease group. These results are invaluable for both the Australian TECs in the study and international clinics involved in similar work, as they confirm how processes involved in moving patients to surgery could be improved. The main finding is that these patients, irrespective of country, all face similar time delays from initial diagnosis to referral to specialist clinics, indicating that improving referral times, and streamlining the pre-operative workup process, are both critical components of improving care for patients with refractory epilepsy.

## Additional files

Additional file 1; Clinical Record Review: Survey Questions. Word document outlines the clinical record review questions (DOCX 16 kb)

Additional file 2; Timeline of visits for all patients in the study. Pdf document showing the clinic activity for all the patients in the study (PDF 56 kb)

## Abbreviations

AED: Anti-epileptic drugs
EEG: Electroencephalogram
GAD7: Generalized Anxiety Disorder screening tool
NNDIE: Neurological Disorder Depression Inventory for Epilepsy
NSW: New South Wales
