# Supplementary material for: Promoting faster pathways to surgery: a clinical audit of patients with refractory epilepsy
Source: BMC Neurol. 2019 Feb 19;19:29. doi: 10.1186/s12883-019-1255-0 (PMC6381714; doi:10.1186/s12883-019-1255-0)
Supplement: Supplementary file 1 — Clinical Record Review: Survey Questions. Word document outlines the clinical record review questions (DOCX 16 kb) [file 12883_2019_1255_MOESM1_ESM.docx]

Q1 Reference number

Q2 Age in years

Q3 Postcode

Q4 Marital Status

- Married/de facto relationship (1)
- Single (2)
- Divorced (3)
- Separated (4)
- Never married (5)
- Not known (6)

Q5 Main Diagnosis

Q6 Secondary Diagnoses

Q7 Date of first (referral) appointment at clinic (dd/mm/yyyy)

Q8 Number of years since epilepsy diagnosed

Q9 Date of first clinic visit during study period (dd/mm/yyyy)

Q10Driving status at first clinic visit during study period

- Eligible to drive (1)
- Not eligible to drive (2)
- Not known (3)

Q11 Employment status at first clinic visit

- Employed full time (1)
- Employed part time (2)
- Unemployed looking for work (3)
- Unemployed not looking for work (4)
- Retired (5)
- Student (6)
- Disabled (7)
- Not known (8)

Q12 Surgery options at first clinic visit

- Surgery not discussed or raised (1)
- Surgery discussed (2)
- Decision made to proceed to surgery (3)
- Decision made NOT to proceed to surgery (4)
- Side effects of surgery discussed (5)
- Referral to surgical team (6)
- Surgery completed (7)
- Post-op visit (8)
- Date of most recent video EEG (dd/mm/yyy) (9)

Q13 Tests ordered at first clinic visit

- Blood tests to review drug levels (1)
- EEGs (2)
- Pre-op work up (3)

Q14HRQoL tests on first clinic visit

- GAD 7 score (1)
- NNDIE score (2)
- Other HRQRoL (please state test and score) (3)
- Was patient seizure free (Y/N) 4)

Q15 Please list epilepsy medications being taken by patient at first clinic visit (please list generic names where possible)

- Carbamazepine (1)
- Phenytoin (2)
- Valproate (3)
- Phenobarbital (4)
- Lamotrigine (5)
- Lacosamide (6)
- Levetiracetam (7)
- Topiramate (8)
- Zonisamide (9)
- Clobasam (10)
- Clonazepam (11)
- Ethosuximide (12)
- Nitrazepam (13)
- Oxcarbazepine (14)
- Others (15)

Q16 Medication management at first clinic visit

- Number of medications changed (1)
- Number of medications where dosage was changed (2)

Q17 Was there a second clinic visit during the study period

- Yes (1)
- No (2)

Skip to end of survey if no further clinic visit was recorded during study period

(Questions 9-17 are repeated for each visit)
