# Supplementary figures and images for: Promoting faster pathways to surgery: a clinical audit of patients with refractory epilepsy
Source: BMC Neurol. 2019 Feb 19;19:29. doi: 10.1186/s12883-019-1255-0 (PMC6381714; doi:10.1186/s12883-019-1255-0)

## Patient pathways in refractory epilepsy

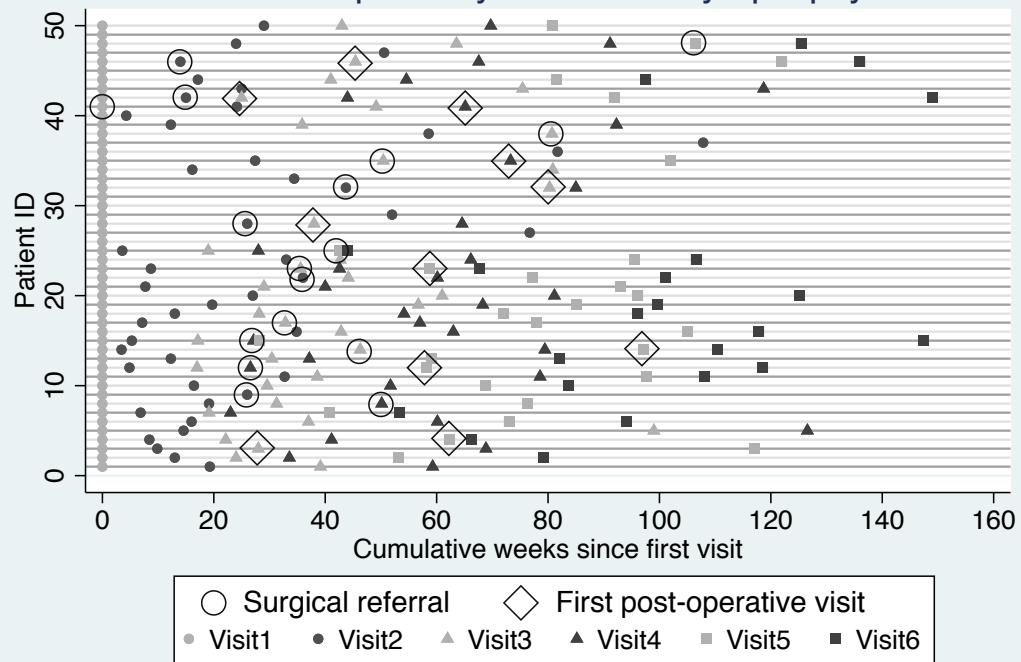

Supplement: Supplementary file 2 — Timeline of visits for all patients in the study. Pdf document showing the clinic activity for all the patients in the study (PDF 56 kb) [file 12883_2019_1255_MOESM2_ESM.pdf]
